# Identification of a ten-long noncoding RNA signature for predicting the survival and immune status of patients with bladder urothelial carcinoma based on the GEO database: a superior machine learning model

**DOI:** 10.18632/aging.202553

**Published:** 2021-02-17

**Authors:** XuDong Mao, ShiHan Chen, GongHui Li

**Affiliations:** 1 Department of Urology, Sir Run Run Shaw Hospital, Zhejiang University School of Medicine, Hangzhou, People's Republic of China; 2 Department of Endocrinology and Metabolism, West China Hospital, Sichuan University, Chengdu, Sichuan, People's Republic of China

**Keywords:** bladder cancer, lncRNA, nomogram, machine learning, immune infiltration

## Abstract

Bladder urothelial carcinoma (BLCA) is recognized to be immunogenic and tumorigenic. This study identified a novel long noncoding RNA (lncRNA) signature for predicting survival for patients with BLCA. A univariate Cox regression model and the random survival forest-variable hunting (RSF-VH) algorithm were employed to achieve variable selection. Ten lncRNAs (LOC105375787, CYTOR, URB1-AS1, C21orf91-OT1, CASC15, LOC101928433, FLJ45139, LINC00960, HOTAIR and TTTY19) with the highest prognostic values were identified to establish the prognostic model. The nomogram integrating the signature and clinical factors showed high concordance index values of 0.94, 0.7 and 0.90 in the three datasets, and the calibration curves showed concordance between the predicted and observed 3- and 5-year survival rates. The risk score based on the 10-lncRNA signature accurately distinguished high- and low-risk BLCA patients with different disease-specific survival(DSS) or overall survival(OS) outcomes, which were stratified according to clinical factors, including T stage and tumour grade. Gene set enrichment analysis identified BLCA-specific biological pathways and enriched functional categories, such as the cell cycle, DNA repair and immune system. Furthermore, the increased infiltration of immune cells in the high-risk group indicated that lncRNA-related inflammation may reduce the survival of BLCA patients.

## INTRODUCTION

Bladder urothelial carcinoma (BLCA) is one of the most prevalent malignancies, with 40 000 additional diagnoses yearly throughout the world [1]. The high malignancy and poor prognosis of BLCA are difficult issues for patients and health professionals. A biomarker, such as a molecule or a clinicopathologic characteristic, has practical value for precise prognosis and individualized treatment. Currently, clinicopathologic characteristics, such as tumour stage (T) and tumour pathological grade (G), remain the prevailing prognostic predictors. However, studies have shown the inadequacy of these clinical features in identifying patients with poor prognosis [2]. Novel molecular biomarkers are expected to serve as prognostic predictors that can help estimate prognosis, select therapeutic strategies and reveal mechanisms of disease.

At present, a growing number of researchers are paying attention to long noncoding RNAs (lncRNAs), which are defined as RNA transcripts longer than 200 nucleotides with limited protein coding potential [3, 4]. Many studies have shown that lncRNAs can promote tumour initiation, development and metastasis by regulating the expression of associated genes at the nuclear (transcription) [5, 6], cytoplasmic (post-transcription) [7, 8] and epigenetic levels [9, 10]. LncRNAs can also serve as competing endogenous RNAs (ceRNAs), interacting with microRNAs [8, 11] and influencing mRNA expression. In addition, abundant evidence suggests that lncRNAs contribute to tumour development by activating immune system processes and immune responses, including antigen release, antigen presentation, immune cell differentiation, immune cell migration, T cell infiltration and the recognition and killing of cancer cells. Since the role of lncRNAs in immuno-oncology is not yet clear, this study investigated the potential interaction between cancer-related lncRNAs and immune checkpoints, as well as the relationship between lncRNAs and immune cell infiltration.

Growing evidence suggests that lncRNAs could function as potential biomarkers or therapeutic targets in many cancer types, especially in carcinomas within the urinary tract [12, 13]. Thus, we believe that lncRNAs are an emerging star in the diagnosis and treatment of various cancers, especially BLCA, because they have specific expression patterns, tumour tissue specificity and stability in circulation. Cancer-related lncRNAs provide novel insight into the complex aetiology and mechanism of the carcinogenesis process. It is reasonable and of great significance to develop a molecular signature based on lncRNAs for identifying the population of BLCA patients with poor prognosis.

## RESULTS

### Determination of prognostic lncRNAs

**Figure 1:**
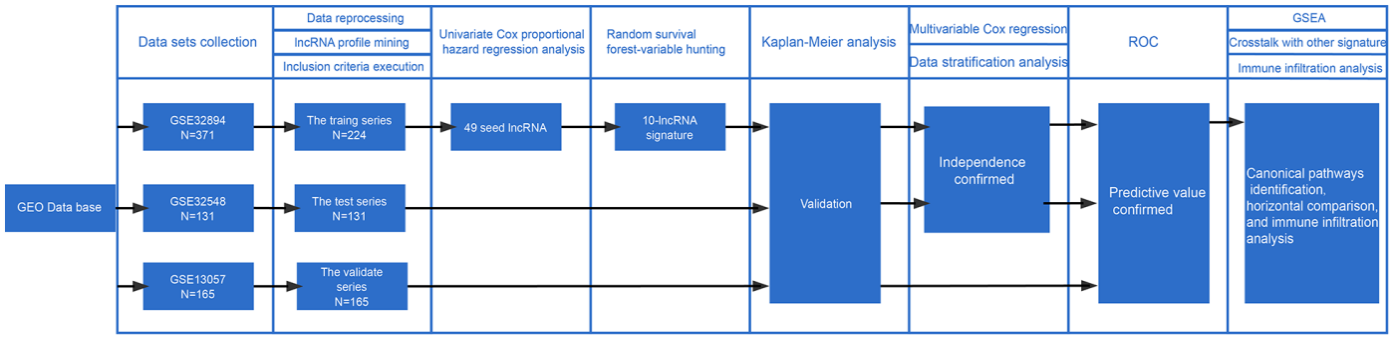
**Workflow of the construction and validation of the signature.** ROC: Receiver operating characteristic; GSEA: gene set enrichment analysis; GEO: Gene Expression Omnibus.

Figure 1 visualizes the identification process. After subjecting the lncRNA expression data to univariable Cox regression analysis by BRB-Array Tools, we identified 49 lncRNAs that strongly correlated with DSS (P<0.01). These 49 lncRNAs and their details, such as regression coefficients, P values and hazard ratios (HRs), are recorded in Supplementary Table 2. RSF-VH was performed on the expression profile consisting of these 49 lncRNAs. With this method, 10 lncRNAs remained for the construction of a prognostic signature for survival prediction. The details of these 10 prognostic lncRNAs, including their P values, regression coefficients, and HRs, are recorded in Table 1. In addition, the out-of-bag (OOB) importance value for each lncRNA is displayed in Table 1 and Supplementary Table 1. We observed that LOC105375787 was the strongest risk factor, while TTTY19 acted as the strongest cancer suppressor. The importance measures the increase (or decrease) in prediction error for the forest ensemble when a variable is randomly permuted in the OOB samples [14, 15]. Supplementary Figure 1 illustrates how the expression value of these genes and patient mortality correlate in random survival trees.

**Table 1:** List of BLCA-specific prognostic lncRNAs.

**Probe Id**	**Symbol**	**Coefficient**	**HR (95% CI for HR)**	**p.value**	**Importance**	**Discription**
ILMN_1884070	LOC105375787	4.4	83 (5.2-1300)	1.80E-03	7.15E-03	uncharacterized LOC105375787
ILMN_1665515	CYTOR	1.2	3.4 (1.5-7.9)	4.60E-03	6.63E-04	cytoskeleton regulator RNA
ILMN_1728403	URB1-AS1	1.2	3.5 (1.5-7.9)	3.00E-03	2.39E-03	URB1 antisense RNA 1 (head to head)
ILMN_1909784	C21orf91-OT1	3.2	24 (3.3-170)	1.60E-03	6.88E-03	C21orf91 overlapping transcript 1
ILMN_1656131	CASC15	0.62	1.9 (1.4-2.5)	7.60E-05	3.09E-03	cancer susceptibility 15
ILMN_1910948	LOC101928433	0.91	2.5 (1.3-4.9)	8.30E-03	1.30E-03	uncharacterized LOC101928433
ILMN_1813179	LINC00960	-0.39	0.68 (0.51-0.91)	9.90E-03	6.83E-04	long intergenic non-protein coding RNA 960
ILMN_2099858	TTTY19	-4.1	0.017 (0.00078-0.37)	9.80E-03	9.15E-04	testis-specific transcript, Y-linked 19
ILMN_1807464	FLJ45139	1.3	3.5 (1.5-8.4)	4.00E-03	1.60E-03	uncharacterized LOC400867
ILMN_1904054	HOTAIR	1.9	6.8 (2.9-16)	1.00E-05	1.00E-02	HOX transcript antisense RNA

Inferentially, positive coefficients implied that higher expression levels of 8 genes including LOC105375787, CYTOR, URB1-AS1, C21orf91-OT1, CASC15, LOC101928433, FLJ45139 and HOTAIR predicted shorter survival. In contrast, negative coefficients implied that higher expression levels of genes including LINC00960 and TTTY19 might predict longer survival. After grouping the patients using the k-means clustering algorithm based on the expression level of each lncRNA, we calculated Kaplan-Meier estimates to display the prognosis related to each prognostic lncRNA.

The overexpression of CASC15, URB1-AS1, FLJ45139, LOC105375787, HOTAIR was related to significantly shortened survival times in patients (log-rank test <0.05). High TTTY19 expression was significantly associated with longer survival in BLCA patients (log-rank test<0.05). The detailed results are shown in Supplementary Figure 2. Additionally, we found that the expression levels of CASC15, FLJ45139, LOC101928433 and C21orf91-OT1 (4 out of 8) were significantly increased in BLCA tissues compared to adjacent tissues or normal mucosas. The expression level of LINC00960 (1 out of 2) was decreased in BLCA tissues compared with normal tissues. However, in contrast to our expectation, lower expression was observed in BLCA tissues. This may be caused by the small sample size of normal bladder mucosa, as only 9 normal bladder tissue samples were included in the analysis (Supplementary Figure 3).

### Risk formula and prognosis

To further investigate the association of this 10-lncRNA signature with BLCA prognosis, a prognostic model was constructed as follows:

∑_i_ Coefficient (lncRNAi) × Expression (lncRNAi), which was

Risk score=

(4.4 × expression level of LOC105375787) +

(1.2 × expression level of CYTOR) + (1.2 × expression level of URB1-AS1) +

(3.2 × expression level of C21orf91-OT1) +

(0.62 × expression level of CASC15) +

(0.91 × expression level of LOC101928433) +

(1.3 × expression level of FLJ45139) +

(1.9 + expression level of HOTAIR) +

(-0.39 × expression level of LINC00960) +

(-4.1 × expression level of TTTY19).

**Figure 2:**
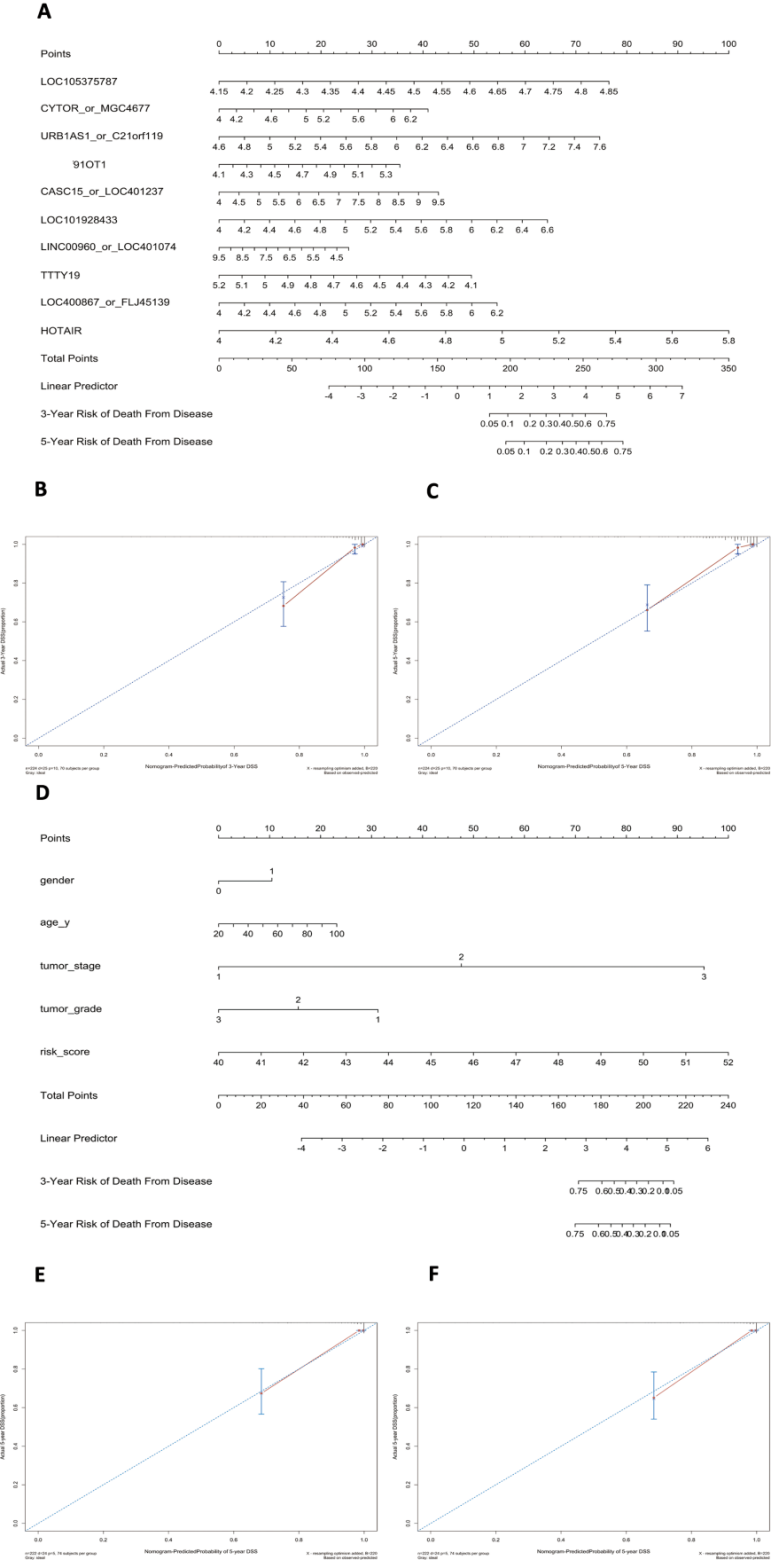
**Nomograms to predict 3- or 5-year DSS in the GSE32894 dataset.** Instructions: locate each characteristic on the corresponding variable axis and draw a vertical line upwards to the points axis to determine the specific point value. Repeat this process. Sum the total points value and locate it on the total points axis. Draw a vertical line down to the 3- or 5-year DSS to obtain the survival probability for a specific bladder cancer patient. (**A**), Nomogram for predicting 3- or 5-year DSS in GSE32894 based on the 10-lncRNA signature. (**B**), Calibration curve for the prediction of 3-year DSS by the nomogram in (**A**). (**C**), Calibration curve for the prediction of 5-year DSS by the nomogram in (**A**). (**D**), Nomogram for predicting 3- or 5-year DSS in GSE32894 based on the 10-lncRNA signature combined with clinical risk factors. (**E**), Calibration curve for the prediction of 3-year DSS by the nomogram in (**D**). (**F**), Calibration curve for the prediction of 5-year DSS by the nomogram in (**D**).

**Figure 3:**
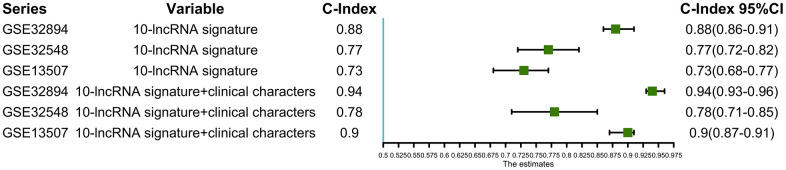
**C-indexes with 95% confidence intervals of the prognostic models.**

Using this formula, we calculated the risk score for each patient (Supplementary Tables 6–9). Defining the median risk score as a cut-off, the patients in the three series were divided into a high-risk subgroup or a low-risk subgroup of the same sample size (N=112, N=66, and N=83 and N=112, N=65, and N=82). Two prognostic nomograms for BLCA patients were successfully constructed to provide a clinically applicable quantitative approach for individual DSS prediction based on the signature and clinical prognostic factors, such as age, sex, grade and T stage (Figure 2A, 2D). The calibration curves in Figure 2B, 2C show a narrow margin between the predicted 3- and 5-year DSS rates and the actual values. The calibration plots in Figure 2E, 2F show excellent agreement between the predicted 3- and 5-year DSS rates and the actual observations. The C-index of the molecular nomogram was 0.88 (95% confidence interval (CI)=0.86-0.91) in GSE32894, 0.77 (95% CI=0.72-0.82) in GSE32548, and 0.73 (95% CI=0.68-0.77) in GSE13507. The C-index of the prognostic model combining the molecular signature with clinical risk factors was as high as 0.94 (95% CI=0.0.93-0.96) in GSE32894, 0.78 (95% CI=0.71-0.85) in GSE32548, and 0.90 (95% CI=0.87-0.91) in GSE13507. The condition number (K value) of the 10-lncRNA signature was calculated in every dataset which confirmed that there was no collinearity among these 10 genes (Supplementary Table 12). These results implied a superior predictive ability of the model, whether based on the 10-lncRNA signature alone or the signature combined with clinical parameters (Figure 3).

**Figure 4:**
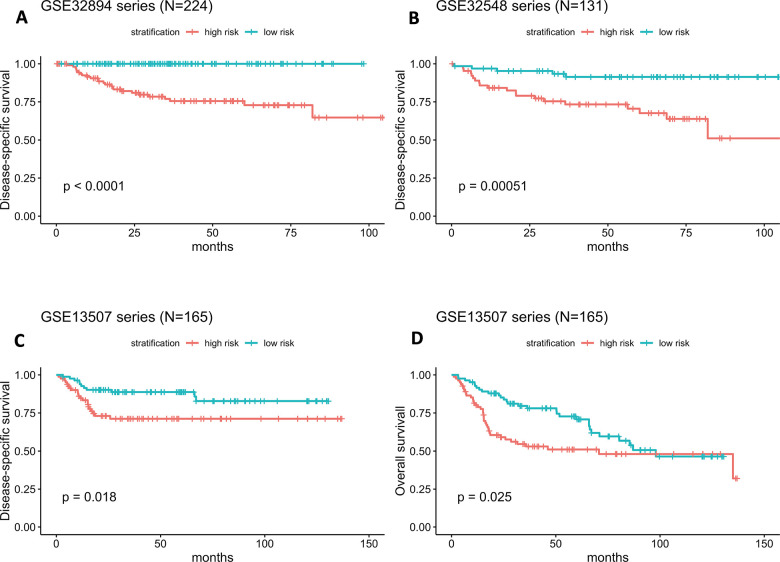
(**A**–**D**) Kaplan-Meier survival curves in subgroups stratified by the risk score. The tick marks on the Kaplan-Meier curves represent censored subjects. The differences between the two curves were assessed by the two-sided log-rank test.

As illustrated in Figure 4A, the Kaplan-Meier analysis of GSE32894 showed that patients with higher risk scores had shorter DSS times than those with lower risk scores (log-rank test P < 0.0001). To further confirm the predictive value of this prognostic model, Kaplan-Meier analyses were performed to validate our signature in two independent external series, GSE32548 and GSE13507. An identical conclusion was obtained in GSE32548, where patients with high risk scores had significantly shorter DSS times than patients in the low-risk group (log-rank test P=0.00051) (Figure 4B). Similarly, in GSE13507, shorter DSS times were observed in the high-risk subgroup (log-rank test P=0.018) (Figure 4C). Even using overall survival (OS) as the follow-up endpoint, the risk score still separated patients with different survival times in the GSE13507 validation series (log-rank test P=0.025) (Figure 4D).

**Figure 5:**
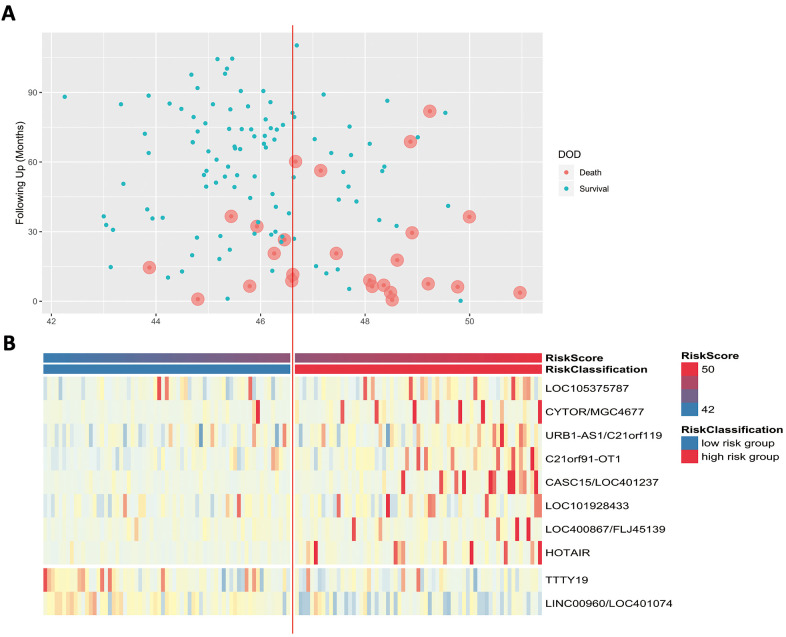
(**A**) Patients’ survival status and DSS time. (**B**) Heatmap of the lncRNA expression profiles. Rows represent lncRNA expression, and columns represent patients. The middle dividing lines represent the median lncRNA risk score cut-off point. The graduated colour, from blue to red, represents the risk score.

Scatter plots and heatmaps in GSE32548 were used to show the relationships among the risk score, the expression levels of ten lncRNAs and death from bladder cancer. Figure 5A shows that patients with higher risk scores tended to have higher mortality. The expression patterns of BLCA-specific lncRNAs were visualized in the heatmaps shown in Figure 5B. Patients with higher risk scores were observed to be more likely to have higher expression levels of risk-related lncRNAs, such as LOC105375787, CYTOR, URB1-AS1, C21orf91-OT1, CASC15, LOC101928433, FLJ45139, and HOTAIR. In contrast, patients with lower risk scores were more likely to have higher expression levels of suppressor lncRNAs (LINC00960 and TTTY19).

### Independence of the 10-lncRNA signature in survival prediction from tumour stage and tumour histopathological grade

A good prognostic biomarker should be independent of prevailing prognostic clinicopathologic factors. The traditional risk stratification of survival mainly depends on histopathological evidence, such as tumour stage and histopathological grade. To confirm the independence and applicability of our 10-lncRNA signature, multivariate Cox regression along with stratification analysis were conducted in GSE32894 and GSE32548.

Figure 6A indicates that both the 10-lncRNA risk score (HR=1.55, 95% CI=1.223–2.0, P <0.001) and tumour stage (HR=12.04, 95% CI=2.665–54.4, P < 0.001) are predictors independent of age, sex and histopathological grade in GSE32894. In GSE32548, using age, sex, tumour stage, histopathological grade and carcinoma *in situ* (CIS) as the covariables, multivariable Cox regression analysis further confirmed that the 10-lncRNA risk score (HR=1.90, 95% CI=1.201–3.0, P <0.007) was a prognostic predictor independent of tumour stage and histopathological grade (Figure 6B).

**Figure 6:**
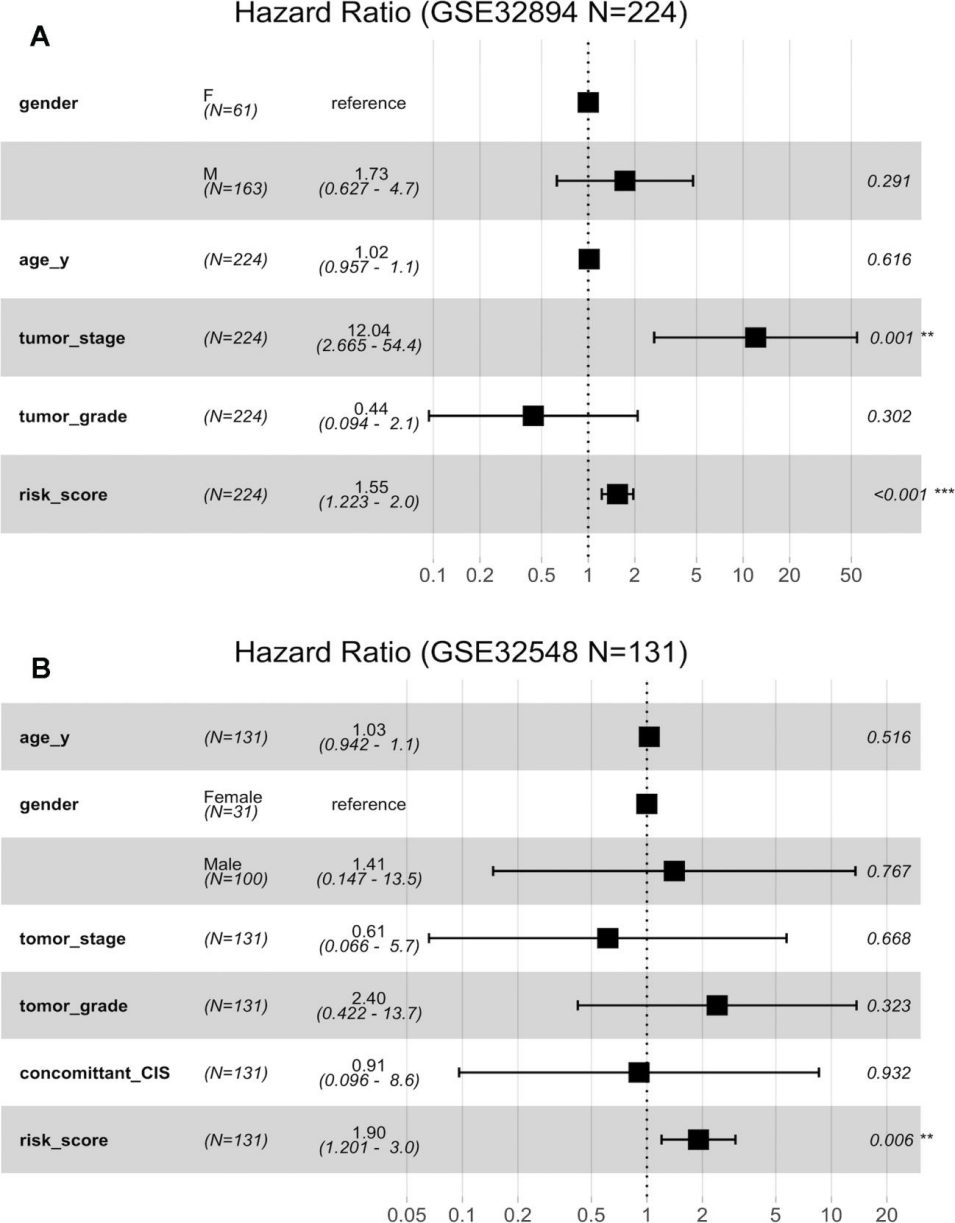
**Forest plots showing the hazard ratios (HRs) with 95% confidence intervals (95% CIs) based on the multivariate Cox regression results.** (**A**) Risk score and tumour stage are dependent of age, sex and histopathological grade in GSE32894. (**B**) Risk score is dependent of sex, age, tumour stage, histopathological grade and concomitant CIS in GSE32894.

**Figure 6:**
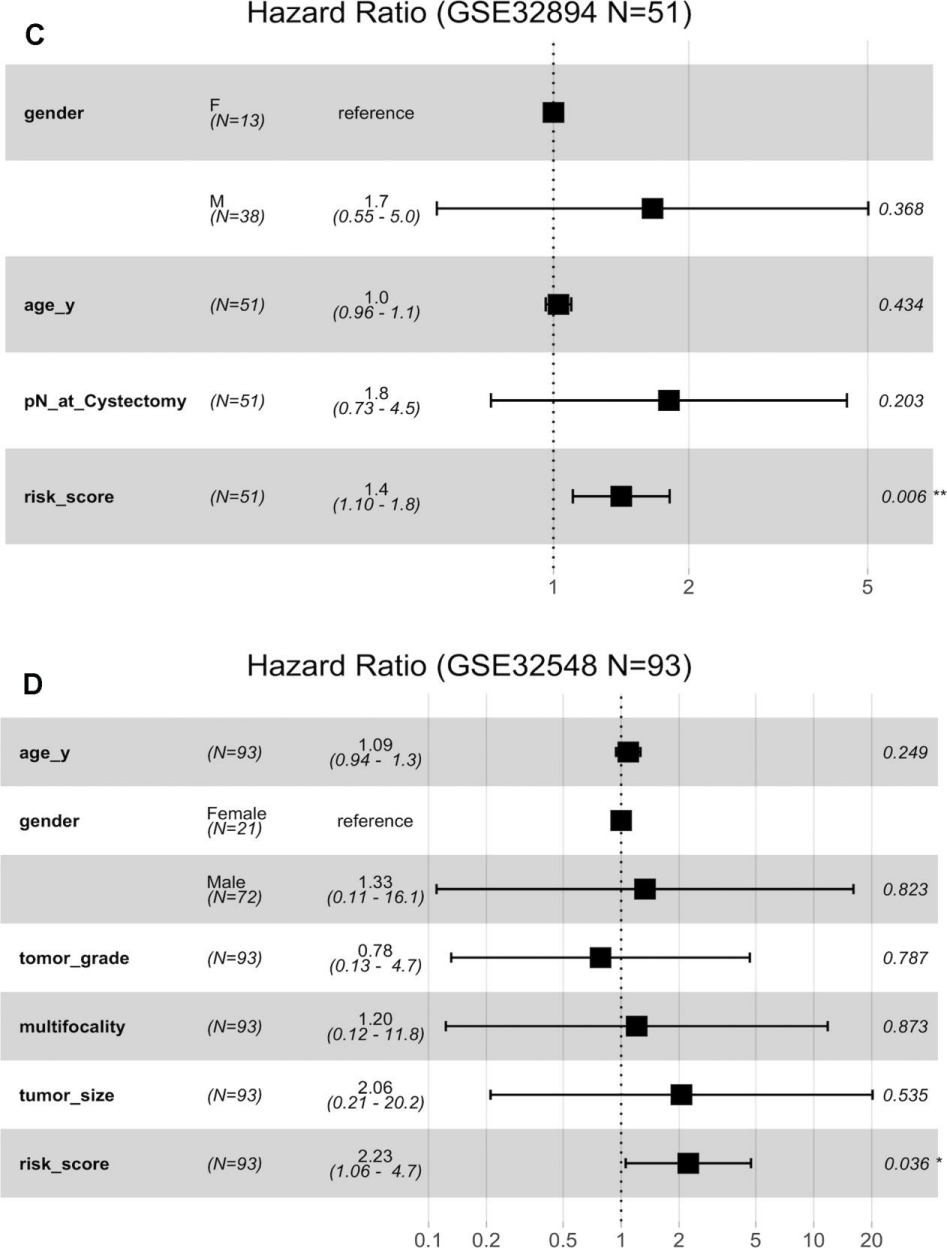
**Forest plots showing the hazard ratios (HRs) with 95% confidence intervals (95% CIs) based on the multivariate Cox regression results.** (**C**) Risk score is dependent of sex, age and pN in patients with cystectomy in GSE32894. (**D**) Risk score is dependent on sex, age, histopathological grade, multifocality and tumour size in patients with NMIBC in GSE32548.

**Figure 7:**
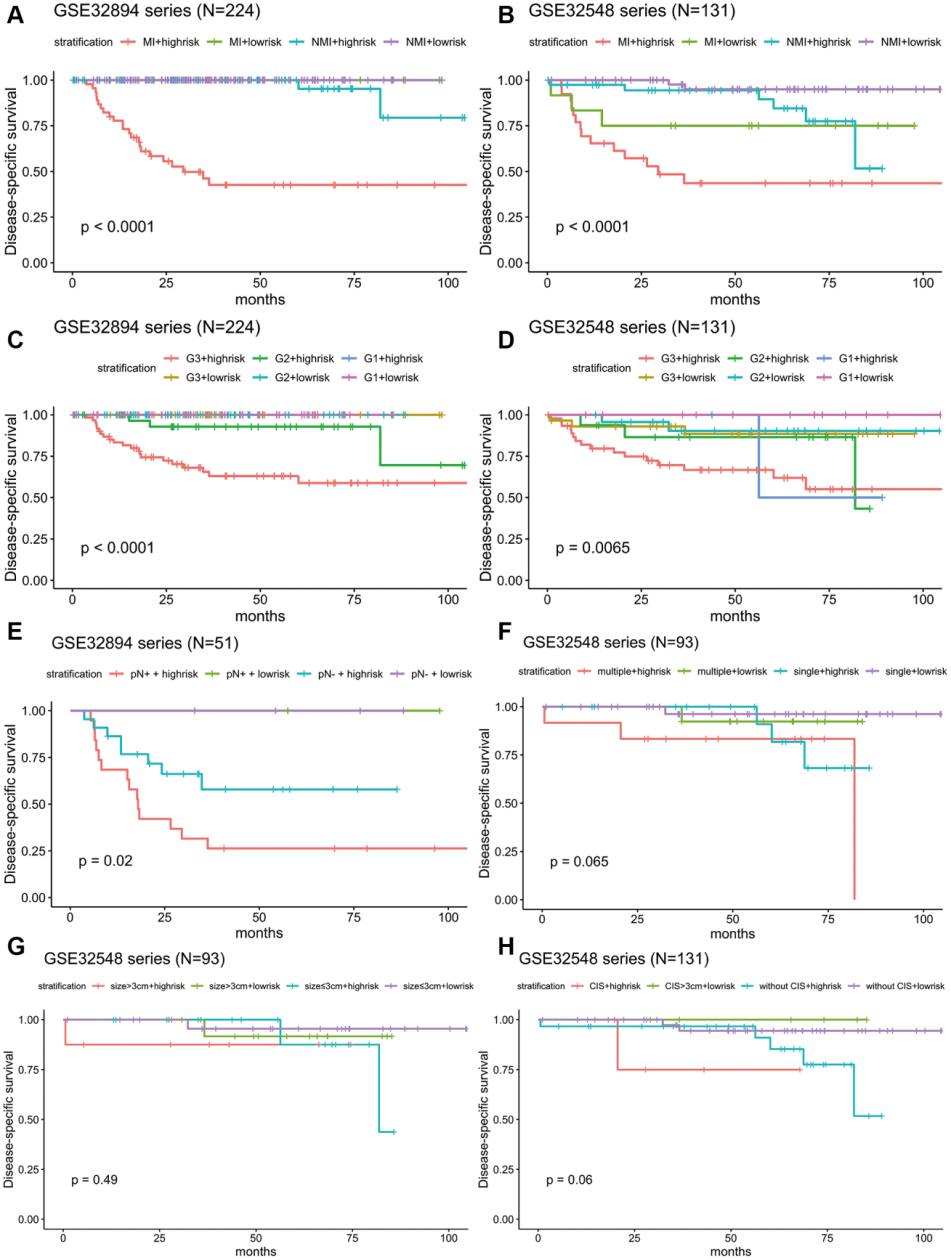
**Kaplan-Meier estimates along with stratification of DSS for patient grouping by the signature.** (**A**) Kaplan-Meier curves for GSE32894 (N = 224), stratified by whether muscle invasion exists (≤T2 or >T2). (**B**) Kaplan-Meier curves for GSE32548 (N = 131), stratified by whether muscle invasion exists (≤T2 or >T2). (**C**) Kaplan-Meier curves for GSE32894 (N = 224), stratified by tumour grade. (**D**) Kaplan-Meier curves for GSE32548 (N = 131), stratified by tumour grade. (**E**) Kaplan-Meier curves for patients with cystectomy in GSE32894 (N = 51), stratified by pN (pN- or pN+). (**F**) Kaplan-Meier curves for patients with NMIUC (<T2) in the GSE32548 test series (N = 93), stratified by multifocality (single or multiple). (**G**) Kaplan-Meier curves for patients with NMIUC (<T2) in GSE32548 (N = 93), stratified by tumour size (≤3 cm or >3 cm). (**H**) Kaplan-Meier curves for GSE32548 (N = 131), stratified by concomitant CIS; the tick marks on the Kaplan-Meier curves represent death from disease. The differences between the two curves were assessed by the log-rank test. MI: muscle invasion; NMI: non-muscle invasion.

Stratification analysis was used to investigate whether the signature could discriminate patients with different prognoses irrespective of the same tumour stage. After stratifying the patients from GSE32894 and GSE32548 into the muscle invasion (≥T2) subgroup or non-muscle invasion (Ta or T1) subgroup, Kaplan-Meier plots showed that for patients in the same tumour invasion subgroup, those with higher risk scores had significantly shorter DSS times than those with lower risk scores (Figure 7A, 7B).

Considering that tumour grade is another primary prognostic factor in BLCA, stratification analysis was conducted to investigate whether the 10-lncRNA signature could accurately predict the prognosis of bladder cancer patients with the same histopathological grade. The patients in both GSE32894 and GSE32548 were classified into three groups (G1, G2 and G3) based on different tumour grades. Kaplan-Meier plots showed that for patients with the same tumour histopathological grade, patients with higher risk scores had significantly lower DSS curves than those with lower risk scores (Figure 7C, 7D).

### Independence of the 10-lncRNA signature in survival prediction from lymph node metastasis, multifocality, tumour size and concomitant CIS

Other clinical and pathological characteristics, such as lymph node metastasis, multifocality, tumour size and concomitant CIS, have been widely recognized to be the prevailing predictors for the prognosis of BLCA patients. Multivariate Cox regression along with stratification analysis were then conducted to determine the prognostic independence of the signature.

Fifty-one patients in the GSE32894 series had available post-cystectomy information, which made it possible to investigate whether the signature is independent of lymph node metastasis. We performed multivariable Cox regression analysis on this cohort, including age, sex, pN at cystectomy, and the 10-lncRNA risk score as the covariables. The analysis showed that the 10-lncRNA risk score (HR=1.4, 95% CI=1.10–1.80, P < 0.007) was the only independent prognostic factor and had a close correlation with DSS (Figure 6C). Kaplan-Meier plots further confirmed that patients with higher risk scores had shorter DSS times than those with lower risk scores, despite having the same pN at cystectomy (Figure 7E).

Clinical information on multifocality and tumour size was available for 93 non-muscle-invasive urothelial carcinoma patients (tumour stage <T2) in GSE32548 by inspection. Multivariable Cox regression analysis performed with the risk score, multifocality and tumour size in this cohort suggested that the 10-lncRNA risk score (HR=2.23, 95% CI=1.06–4.7, P < 0.037) was the only independent prognostic factor (Figure 6D). Since the sample size of non-muscle-invasive urothelial carcinoma was small, Kaplan-Meier plots generated indistinctive Kaplan-Meier curves (P =0.065 and 0.49) among the stratifications comparing multifocality and tumour size. However, patients with higher risk scores were still more likely to have a poor prognosis, which was indicated by the red or green curves in Figure 7F, 7G.

When compared with concomitant CIS, age, sex, tumour stage and tumour grade, the 10-lncRNA risk score (HR=1.90, 95% CI=1.201–3.0, P <0.007) was determined to be a survival predictor independent of concomitant CIS according to previous results (Figure 6B). Kaplan-Meier plots with stratification showed that the 10-lncRNA signature was able to classify patients who had different survival times and the same concomitant CIS status (Figure 7H) at some level, though the P value did not reach the threshold (log-rank test P=0.06>0.05).

### The prognostic value of the 10-lncRNA signature

To determine the predictive power of this 10-lncRNA signature, ROC curves were employed, and AUC values were calculated. AUCs were compared between the signature and other prevailing prognostic predictors.

**Figure 8:**
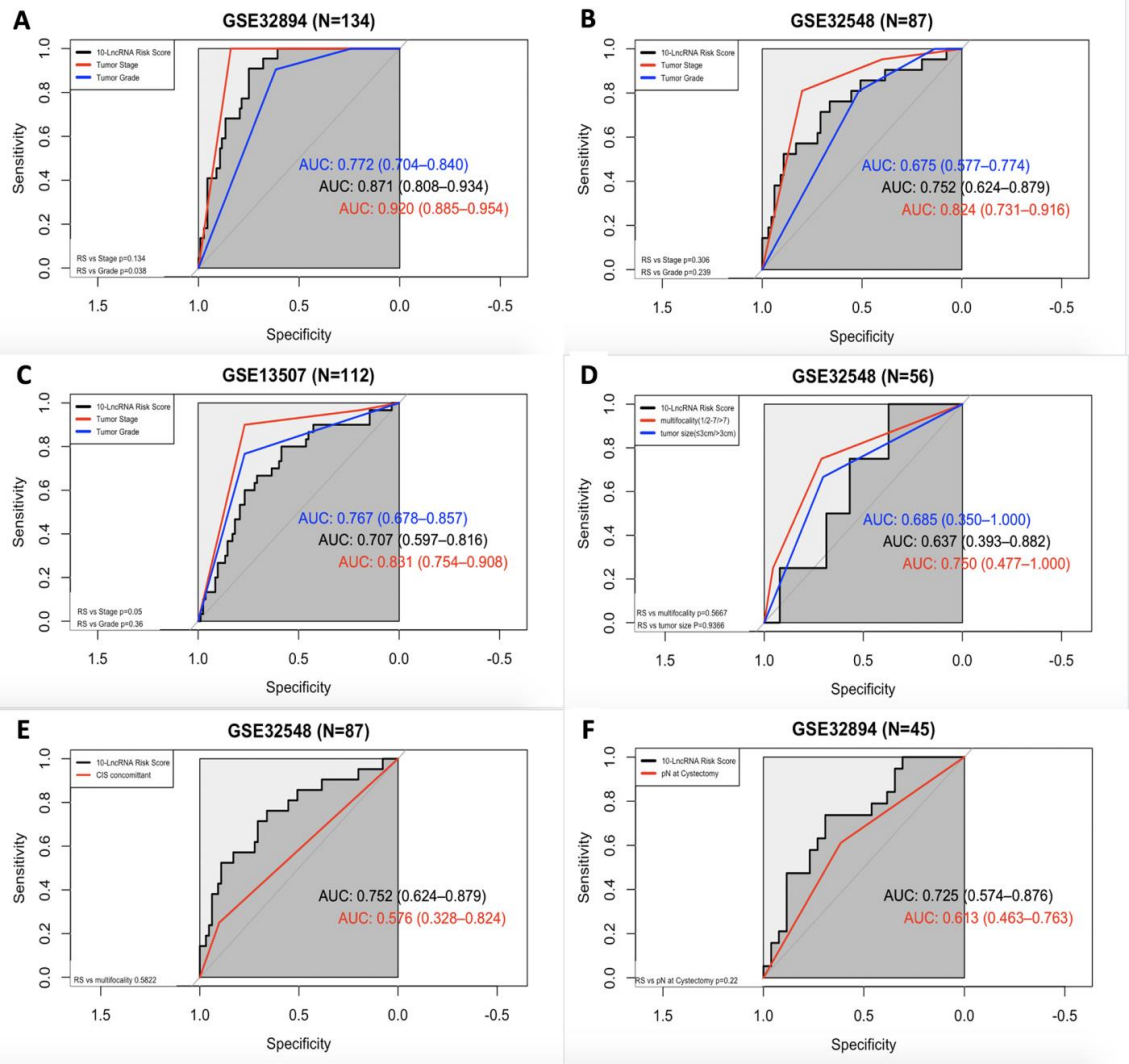
**ROC curves showing the prognostic performance of the 10-lncRNA signature compared with that of different prognostic predictors.** (**A**) Comparison of tumour stage and tumour grade in GSE32894. (**B**) Comparison of tumour stage and tumour grade in GSE32548. (**C**) Comparison of tumour stage and tumour grade in GSE13507. (**D**) Comparison of multifocality and tumour size in patients with NMIUC from GSE32548. (**E**) Compared with concomitant CIS in GSE32548. (**F**) Comparison with pN in patients with MIUC from GSE32894. NMIUC: non-muscle-invasive urothelial carcinoma; MIUC: muscle-invasive urothelial carcinoma.

In all three independent series, the 10-lncRNA signature demonstrated a discriminatory ability for predicting DSS, with AUC values of 0.871 (95% CI=0.808–0.934) in GSE32894 (Figure 8A), 0.752 (95% CI=0.624–0.879) in GSE32548 (Figure 8B) and 0.707 (95% CI=0.597–0.816) in GSE13507 (Figure 8C). As shown by our data, this 10-lncRNA signature had robust sensitivity and specificity to potentially become a superior prognostic biomarker.

In GSE32894, as shown in Figure 8A, both the 10-lncRNA risk score (AUC=0.871) and tumour stage (AUC=0.920) showed a high predictive performance with no significant difference (P=0.134). Compared with histopathological grade, the 10-lncRNA risk score showed greater prognostic potential (0.871 versus 0.772, P=0.0038). In GSE32548, as shown in Figure 8B, since the P value of DeLong's test did not reach the threshold, we cannot tell whether the risk score has a greater capability to predict survival than tumour stage (0.752 versus 0.824, P=0.306) or histopathological grade (0.752 versus 0.675, P=0.239). However, with an absolute AUC value estimated to be 0.752, the prognostic value of this 10-lncRNA signature was still considered remarkable. In GSE13507, as presented in Figure 8C, despite a smaller AUC (AUC=0.707), which was still estimated to exceed 0.70, there was no significant difference between the signature and tumour stage or histopathological grade (P=0.05 and P=0.36, respectively).

ROC curves were also calculated for the 10-lncRNA risk score, concomitant CIS, lymphovascular invasion, multifocality and tumour size. Compared with tumour multifocality and tumour size, as presented in Figure 8D, the AUC of the 10-lncRNA risk score was roughly the same (0.637 versus 0.750; 0.637 versus 0.685, 95% CI=0.393–0.882, P=0.5667; P=0.9366). As shown in Figure 8E, the AUC of the 10-lncRNA risk score was estimated to be greater than 0.70 in GSE32548, which was larger than that of concomitant CIS despite P>0.05 (0.752 versus 0.576, 95% CI=0.624–0.879, P=0.5822). Compared with lymphovascular invasion, as shown in Figure 8F, the 10-lncRNA risk score performed as a relatively good predictor and had a larger AUC (0.725 versus 0.613, 95% CI=0.574–0.876 P=0.22), albeit with no significance observed.

### Relationship among the 10-lncRNA signature, potential therapeutic target signature and immune-checkpoint blockade (ICB) immunotherapy-related signature

A growing body of evidence highlights the key role of immune regulation in BLCA, involving some immune checkpoints, such as CTLA4, PD-1, and PD-L1. In addition, several genomic alterations, such as HER-2 (ERBB2), ERBB3 and FGFR3, which were included in this study, have been identified to be amenable in principle to therapeutic targeting and were reported to be associated with advanced BLCA in previous studies.

**Figure 9:**
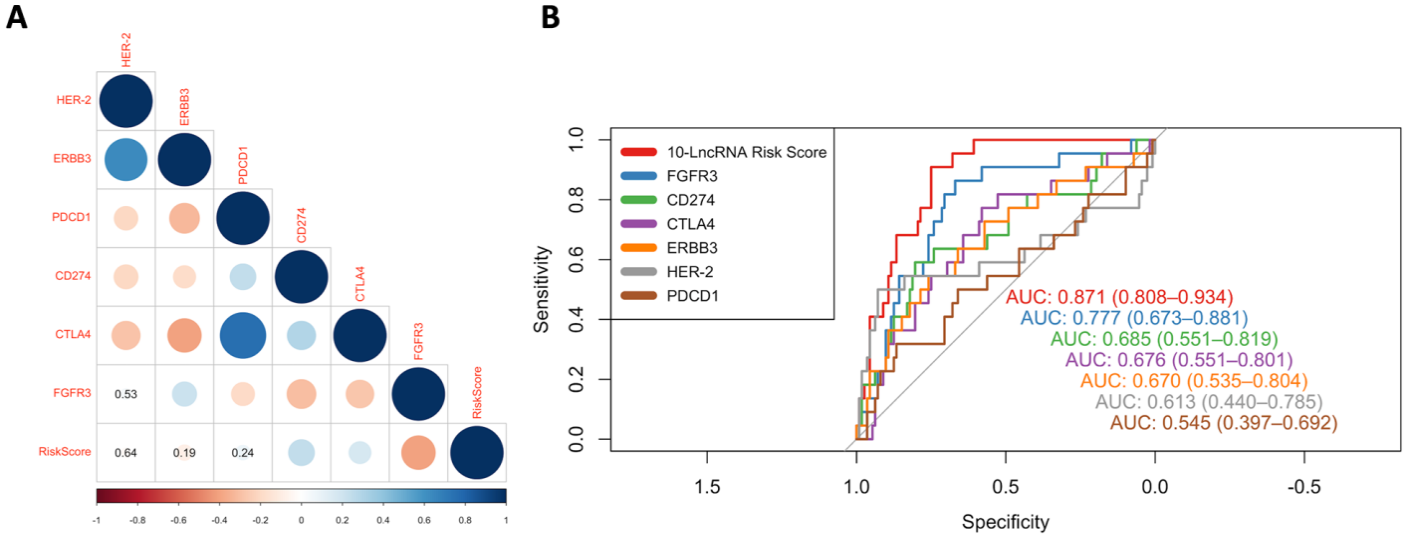
**ROC curves and correlation analysis of different prognostic signatures.** (**A**) Correlation analyses among known immune checkpoints, potential therapeutic targets and the risk score. The circle size represents the P value, and the colour represents the correlation coefficient. Blue indicates a positive correlation, and red indicates a negative correlation. (**B**) ROC curves show the sensitivity and specificity of our 10-lncRNA signature and other known biomarkers in predicting the DSS of patients from GSE32894.

Here, we compared our 10-lncRNA signature with these biomarkers to determine their prognostic value and potential interconnection. Pearson correlation coefficients demonstrated that the 10-lncRNA signature had a negative correlation with FGFR3 and a positive correlation with CD274 (PD-L1) and CTLA4 (P<0.01) (Figure 9A). The AUC values of the ROC curves were calculated for each biomarker. The ROC curves showed that the 10-lncRNA signature had the largest AUC (estimated to be 0.871) compared with these therapeutic targets and immune checkpoints (DeLong's test P < 0.05) (Figure 9B). Assuming these molecules are all promoters regulating cancer development, the results indicated that our lncRNA signature had better stability and reliability in predicting the DSS of patients with BLCA and implied a closer relation between the cancer and the signature.

### Determination of disease-related pathways

**Figure 10:**
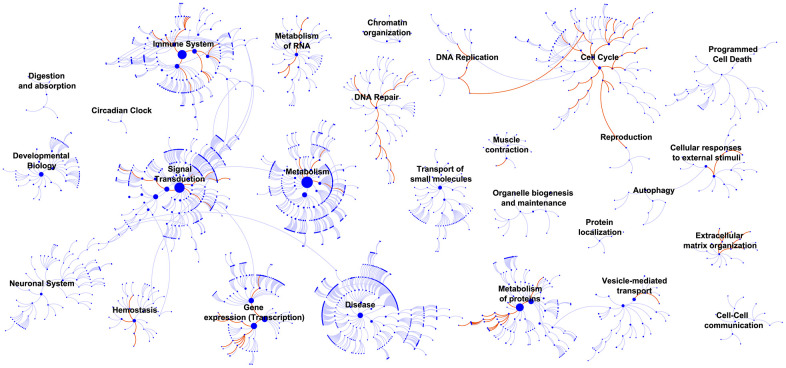
**GSEA identified biological processes that correlated with the 10-lncRNA signature.** Enriched canonical pathways (FDR < 0.01) were mapped in the whole pathway overview. Each dot represents a function, and each line indicates a pathway.

After grouping the patients in GSE32894 into two groups by the median risk score, the whole-genome expression profiles were subjected to analysis with GSEA 3.0 software to screen for pathways related to the signature. Figure 10 visualizes the enriched canonical pathways that were identified as “enriched (FDR < 0.01; P<0.05)” and maps them in a whole pathway overview. The immune system, DNA repair and cell cycle were observed to be the top three enriched pathways. Activation of E2F1 target genes at G1/S, G1/S-specific transcription, activation of the pre-replicative complex, activation of ATR in response to replication stress, and scavenging by class A receptors were the top 5 downregulated canonical pathways. RNA polymerase I promoter clearance, glucuronidation, RNA polymerase I transcription initiation, RNA polymerase I promoter escape, and synthesis of glycosylphosphatidylinositol (GPI) were the top 5 upregulated canonical pathways. Supplementary Table 10 contains all these significantly enriched canonical pathways.

### Analysis of the immune status of the high-risk and low-risk populations

**Figure 11:**
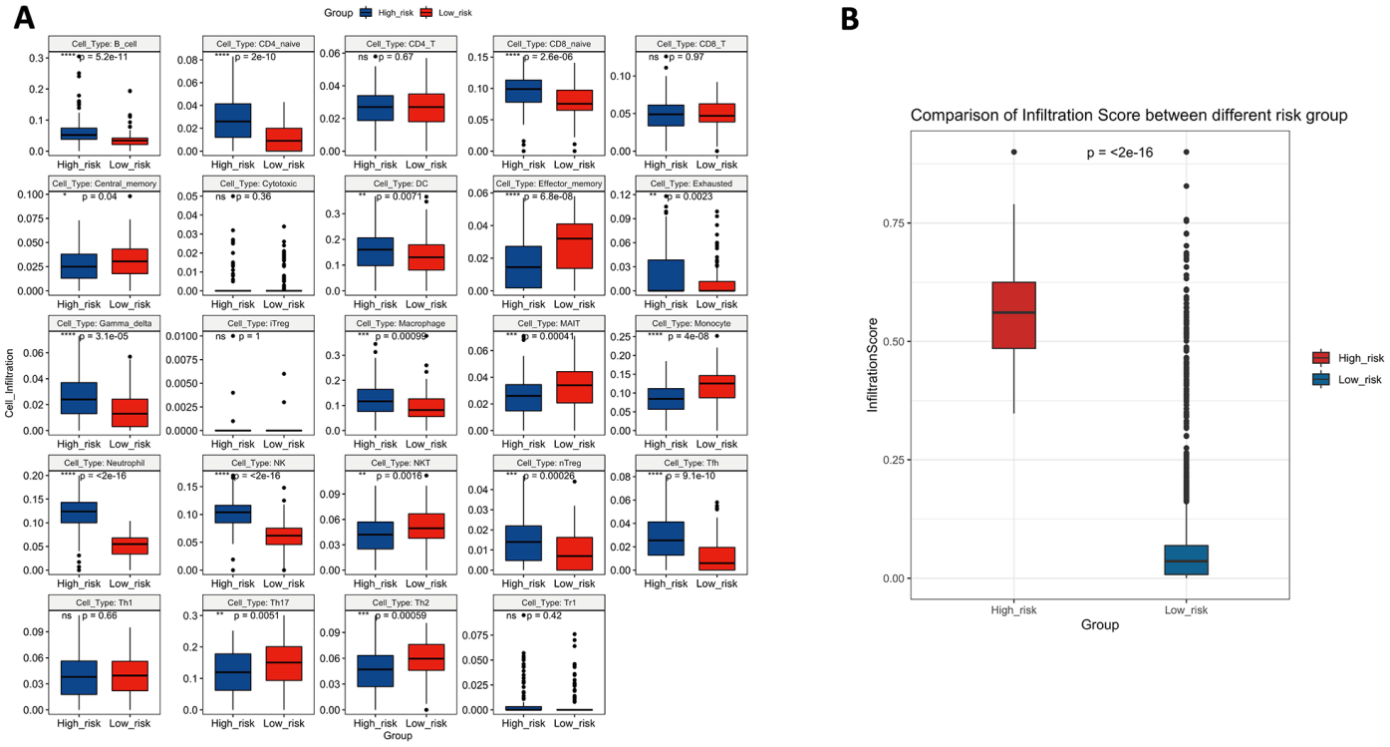
(**A**) The difference in tumour-infiltrating immune cells among risk groups as defined by the 10-lncRNA prognostic signature. (**B**) The difference in infiltration score among risk groups as defined by the 10-lncRNA prognostic signature.

To verify whether the 10-lncRNA signature can reflect the status of the tumour immune microenvironment, we analysed the relationships between the signature and immune cell infiltration. Figure 11A shows that the proportions of infiltrating B cells, naive CD4+ T cells, naive CD8+ T cells, dendritic cells, exhausted T cells, gamma delta T cells, macrophages, monocytes, neutrophils, NK cells, natural regulatory T cells, and follicular helper T cells were significantly enriched in the high-risk group (P<0.05). Central memory T cells, effector memory T cells, mucosal-associated invariant T cells, NKT cells, T helper 17 cells, and T helper 2 cells were significantly enriched in the low-risk group (P<0.05). The infiltration score retrieved from the ImmuCellAI resource is a reflection of the overall tumour immune microenvironment. Figure 11B shows that tumours in the low-risk group have higher infiltration scores, which suggests the connection between the prognostic signature and the inflammatory environment of bladder tumours (P<0.05).

## DISCUSSION

Recently, by combining clinical survival outcomes with data from high-throughput genomic technologies, microarrays have emerged as a popular way of revealing disease characteristics at the molecular level. Although the potential for discovery can be far greater when using such genome-wide data, the high-dimensionality, batch effect and various platforms of this kind of data pose challenging statistical issues. Currently, Student’s t-test remains the popular method to obtain differentially expressed genes (DEGs) from microarrays; however, the lists of DEGs for a given disease indication identified by different studies are highly unstable, and BLCA is no exception [16]. The inconsistencies among gene signatures from different studies have been attributed to many sources, including limited numbers of samples, disease heterogeneity, subtle gene expression variation undetected by current feature selection method, etc. [17]. This may be part of the reason why there is no approved biomarker for the diagnosis and treatment of BLCA and why the mechanism underlining BLCA pathology remains largely unknown. The absence of a suitable model with generalization and precision remains a challenge for bioinformaticians. However, of course, for a predictive model, generalization is far more important than precision. The positive performance of a valuable biomarker in multiple cohorts is considered worthy of further translational research.

To address three datasets with high throughput, multiplatform and multidimension data from different sources, we employed an artificial intelligence method, RFS-VH, to achieve variable selection [14, 18]. It has the following advantages:

(1) RSF can handle very high-dimensional data (data with many features);

(2) When there is a large amount of missing data, RSF can still give accurate predictions;

(3) RSF has an anti-overfitting ability. Decision tree voting reduces the risk of overfitting;

(4) RSF is very stable and accurate. Wrong predictions will only be made when more than half of the base classifiers have errors. When one of the data points causes bias, the entire algorithm will not be greatly affected.

This paper exemplified the great power of RSF in screening potential genes that may have been overlooked in other previous bioinformatics analyses. All significant lncRNAs were modelled into a risk formula. The integration of all molecular predictors into one model is more stable than a single-factor prediction [19]. Nomograms were established to visualize the integrated risk score system. The C-index was estimated to be extremely high; for example, in the external validation set, it was as high as 90% with the signature and clinical factors. Our machine learning-based model has been the best performer ever of its type [20–22]. Superior C-indexes and calibration curves suggested that the risk model had an outstanding ability to predict BLCA survival, whether based on the signature alone or the signature combined with clinical factors. The C-index values of the two models were very close, which also indicated the excellent predictive ability of the molecular biomarker model because it could almost replace the prediction by clinical factors. Kaplan-Meier estimates, multivariate Cox regression analysis and ROC curves, some embedded with stratification analysis, determined the independent prognostic value of the signature. All these analyses indicated that the signature had stable accuracy for predicting the DSS or OS of BLCA patients and was even more robust than age, sex, tumour stage, histological grade, lymph node metastasis, multifocality, tumour size and concomitant CIS. Traditionally, tumour grade and tumour stage have been regarded as the top survival predictors [23]; however, our calculations in multivariate analysis and ROC analysis found a prognostic deficiency of BLCA.

We found that LOC105375787, CYTOR, URB1-AS1, C21orf91-OT1, CASC15, LOC101928433, FLJ45139, and HOTAIR acted as oncogenes, while LINC00960 and TTTY19 acted as suppressors.

However, we did not observe that every single lncRNA in the signature was significantly related to prognosis. Batch effects and a limited number of samples may account for this difference. We believe that their association with DSS may occur directly through carcinogenic roles or as tumour progression-associated bystanders, and their diverse roles suggest that they may function as synergistic facilitators of progression (Supplementary Table 13) [24]. LOC105375787, LOC101928433, FLJ45139 and URB1-AS1 were discovered for the first time as cancer promotors. Except for HOTAIR, all 9 prognostic genes were reported for the first time as BLCA-specific prognostic genes. Cytoskeletal regulator RNA, known as CYTOR, is aberrantly overexpressed in various highly malignant cancers, including breast cancer, colorectal cancer, gastric cancer and non-small cell lung cancer cells [25, 26]. The lncRNA C21orf91-OT1 was significantly increased in foetal growth restriction, although it has no cancer-associated evidence published yet [27]. Cancer susceptibility candidate 15, termed CASC15, located on chromosome 6p22, was reported to suppress the aggressive tumour progress; and the overexpression of its short isoform (CASC15-S) was found to inhibit neuroblastoma progression and increase patient survival [28]. In another study, it was reported that CASC15 was a frequently gained genomic segment in metastatic melanoma tumours [29]. According to He Tianyu et al, CASC15 was confirmed to be overexpressed in 59% of hepatocellular carcinoma tissues compared with corresponding adjacent normal tissues and is believed to act as a tumour promoter in tumorigenesis [30]. Long intergenic non-protein coding RNA 960 (LINC00960) was found to play a suppressor role in BLCA in the present study and was found to be involved in idiopathic pulmonary fibrosis and pancreatic cancer as a positive regulator in the studies by Hadjicharalambous Marina R et al. and Wu Yingcheng et al. [31, 32]. Testis-specific transcript Y-linked 19 lncRNA, also called TTTY19, is considered a male-specific lncRNA, but virtually no existing study has described it. Similar to the downregulated expression of TTTY19 in BLCA in the present study, Lai I-Lu et al. suggested that lower expression levels of another testis-specific transcript Y-linked RNA, termed TTTY15, were related to non-small cell lung cancer proliferation and metastasis [33]. Of particular note, HOTAIR, which is well characterized in many primary tumours, was included in our 10-lncRNA signature. Interacting with polycomb repressive complex 2 (PRC2), HOTAIR acts as a powerful oncogene in BLCA [9, 10, 34]. Furthermore, the expression level of HOTAIR could be suppressed by inhibitors to limit the metastatic potential of cancer, which also indicated that HOTAIR has therapeutic value as an efficient drug target [35].

In GSEA, top-ranked pathways, such as RNA polymerase I promoter clearance, RNA polymerase I transcription initiation, and RNA polymerase I promoter escape, were found in the cell cycle functional categorization [36, 37], suggesting that the 10 lncRNAs exacerbated BLCA by influencing proliferation. Xifeng Wu et al. reported that individuals with a higher number of altered alleles in DNA repair and the cell cycle are at an increased risk of developing bladder cancer, and these genetic effects were found to be significantly related to smoking. Thus, we can speculate that the 10 BLCA-specific lncRNAs could regulate cancer at the epigenetic level by the same pathway [38]. As BCG became the earliest immunotherapy regimen for BLCA, it is clear that BLCA is an immunogenic cancer with components of the immune system successively activated [39]. Intriguingly, GSEA highlighted the enriched pathways of the immune system and suggested the potential value of the signature for inferring the immune characteristics of BLCA.

Inspired by GSEA, to further support our finding, we used ImmuCellAI to obtain a high resolution of the immune infiltration landscape and estimate the abundance of 24 immune cells in the high-risk and low-risk groups. An increased abundance of B cells, naive CD4+ T cells, naive CD8+ T cells, dendritic cells, exhausted T cells, gamma delta T cells, macrophages, monocytes, neutrophils, NK cells, natural regulatory T cells, and follicular helper T cells was proven to promote BLCA aggressiveness, and a reduced abundance of central memory T cells, effector memory T cells, mucosal-associated invariant T cells, NKT cells, T helper 17 cells, and T helper 2 cells was observed in the high-risk group, indicating a “hot” tumour immune microenvironment in BLCA tissues. A higher infiltration of inflammatory cells indicates an enhanced immune environment; similarly, significantly lower immune infiltration has been demonstrated to facilitate BLCA progression. For example, Hartana C A et al reported that a high number of memory T cells in tissue may infiltrate into tumours of lower stage [40]. Another previous study showed that reduced Th17-related cytokines were significantly lower in BLCA patients than in healthy people, and our data showed a similar result; the low-risk group had a higher level of Th17 infiltration [41]. Agarwal A et al. found that Th2 cell expression was significantly lower in BLCA tissues than in healthy tissues, and this finding corresponded to our data [42]. In summary, the 10-lncRNA signature can evaluate the enhancement of the tumour immune microenvironment and is correlated with BLCA progression.

Interestingly, several previous studies similarly reported that some of these noncoding RNAs were involved in the regulation of the immune response. For example, Wang C et al. reported that CYTOR can enable B cell growth and transformation, which is consistent with our result that B cells showed significant infiltration in high-risk individuals [43]. Li SS et al. found C21orf91-OT1 to be dominated by functional processes of the immune response [27]. Yin Y et al. reported that CASC15 could epigenetically silence the expression of the immunomodulatory molecule programmed cell death 4 (PDCD4) and facilitate proliferation and invasion in melanoma cells [44]. To determine the prominent prognostic value of this immune-related-lncRNA, ROC curves were used to compare the AUC values of the risk score, immune checkpoints (PD-1, PD-L1 and CTLA4) and potential therapeutic targets (HER-2, ERBB3 and FGFR3). The results showed that our immune-related-signature did have a higher predictive performance than all the other signatures that were reported to have the potential to serve as predictors of BLCA survival [45–49].

## CONCLUSIONS

In conclusion, this study generated a lncRNA signature that can not only predict BLCA patient survival outcomes but also reflect the immune status of BLCA at some levels. The model built based on this BLCA-specific lncRNA signature has an impressively high accuracy that indicates a high clinical translation value.

## MATERIALS AND METHODS

### Material preparation

We downloaded three whole-genome expression microarray data series from the Gene Expression Omnibus (GEO) (http://www.ncbi.nlm.nih.gov/geo/). All data series were included based on the following criteria: 1. public expression data generated for BLCA were obtained; 2. the same manufacturer platform was used (in this paper, the following Illumina human expression beadchip platforms were used: GPL6947 and GPL6102); and 3. raw nonnormalized data and matched clinical data with follow-up information were obtained. Ultimately, GS32894, GSE32548 and GSE13507 were included in the present study after an initial quality check. The clinical information of the samples included in this study from GSE32894, GSE32548 and GSE13507 was recorded in Supplementary Tables 3–5. The information relative to the selected datasets has been organized in Supplementary Table 11. The BLCA samples in GSE32894 were defined as a training series (N=224), and the samples in GSE32548 were defined as an independent test series (N=131). In addition, 165 samples from GSE13507 were defined as an external validation series (N=165). In this study, we set two independent external datasets (GSE32548 and GSE13507) as verifiable groups rather than separating them because multiple independent validation datasets with larger sample sizes could reduce disease heterogeneity and improve model stability.

### Data pre-processing and lncRNA mining

All bioinformatic approaches used in the present article were conducted with R software version 3.5.2 [50]. The raw nonnormalized expression data of the three series that were directly downloaded from the GEO database underwent noise correction and quantile normalization using the “limma” package in R. This method also log2 transformed the expression values and filtered control probes, leaving only the regular probes [51].

In GEO or other databases, we failed to find a microarray specifically designed for lncRNAs with the requisite clinical information. Therefore, by annotating the probes, we separated a lncRNA profile from total RNA expression data. The gene sequence IDs obtained from the GPL6947 platform mainly contained Unigene ID and Refseq_ID. The “org.Hs.eg.db” package in R was used to obtain three maps of the correspondence between diverse gene sequence IDs, including Unigene ID - Entrez ID, Refseq_ID - Entrez_ID and Entrez ID - Ensembl ID. According to these maps, each Unigene ID and Refseq_ID was mapped to the corresponding Entrez ID. After that, the Entrez ID was further matched to the Ensembl ID, which could be annotated into the corresponding gene type using annotation information obtained from the Ensembl genome database (http://www.ensembl.org/). Ultimately, 11 types of lncRNAs were filtered from the multigene types. Noncoding RNA, lincRNA, processed transcript, TEC, bidirectional lncRNA, macro lncRNA, sense overlapping, sense intronic, retained intron, antisense lincRNA, and 3 prime overlapping ncRNAs were finally retained to generate a re-annotated lncRNA profile containing 911 lncRNAs.

### Bioinformatics analysis

The GSE32894 series, which had a larger sample size (N=224), was used to determine the candidate prognostic lncRNAs. Through univariate Cox regression analysis of the GSE32894 series, lncRNAs that were significantly associated with disease-specific survival (DSS) were screened out as seed lncRNAs for further analysis (P<0.01). The random survival forest-variable hunting (RSF-VH) method was employed to identify the optimal prognosis-related lncRNAs. Here, we used the method with the value argument “nsplit” set to 10, “nrep” set to 100, and “nstep” set to 5, with 1000 trees grow and the k value set to 5 [18, 52]. The expression levels of the most valuable lncRNAs were compared among BLCA tissue (n=165), cancer-adjacent tissue (n=58) and normal bladder mucosa(n=9) in GSE13507.

Weighted by the regression coefficient, a risk score formula was constructed based on the expression level of each candidate lncRNA. Applying this formula, a risk score was calculated for every patient. By checking whether the risk score was greater than the median, the patients were separated into a high-risk (higher score) subgroup or a low-risk (lower score) subgroup. Kaplan-Meier estimates and the log-rank test were employed to assess the difference in prognosis between the two subgroups. Multivariate Cox regression analysis was conducted to determine predictive factors for BLCA prognosis and their independence from other clinical predictors. Using the “rms” package in R, two prognostic nomograms were established to predict the 3-year and 5-year DSS in GSE32894. One was based on the combination of the lncRNA signature with clinical factors, and the other was based on this signature alone. The predictive abilities of these nomograms were assessed with the concordance index (C-index) and calibration curves to compare the model-predicted values and actual observations of DSS. Receiver operating characteristic (ROC) curves were used to determine the prognostic value of the risk score, and the area under the curve (AUC) was also calculated for comparison with other clinical predictors using DeLong's test. In this method, patients having shorter DSS than the median DSS was labelled as positive and patients having longer DSS than the median DSS was labelled as negative. Patients surviving shorter than the median DSS time at the end of follow-up were excluded unless death had been observed. Correlation analysis using Pearson correlation coefficients was used to explore the associations among the 10 lncRNA-based risk scores and the expression levels of immune checkpoints and several potential therapeutic targets (P<0.01).

P < 0.01 was considered statistically significant in univariate Cox regression analysis and Pearson correlation analysis. In the rest of the methods, P<0.05 was considered statistically significant.

### Gene set enrichment analysis (GSEA)

GSEA was conducted between the high-risk subgroup and low-risk subgroup from the GSE32894 series to identify potential cancer-related pathways. GSEA software V3.0 was employed to perform this analysis. Canonical representations of the biological process set, termed “c2.cp.v6.1.entrez.gmt” (1030 gene sets), were used in GSEA. The enrichment results were visualized in Cytoscape software V3.2.1. Dysregulated pathways were mapped in terms of biological processes. Gene sets with false discovery rate (FDR) values <25% and P<0.05 were termed “enriched” after performing 1000 random sample permutations.

### ImmuCellAI analysis

ImmuCellAI (http://bioinfo.life.hust.edu.cn/web/ImmuCellAI/) is an emerging tool used to estimate the abundance of 24 immune cells and overall infiltration scores based on a gene expression data set. The infiltrating data of the high-risk group in GSE32894 (N=112) and the low-risk group in GSE32894 (N=112) were obtained from the ImmuCellAI website. The abundance of each type of immune cell was then tested to detect the differences between the classified prognostic risk groups using the Wilcoxon test, and the results were visualized by a box plot. P<0.05 was considered statistically significant.

## Supplementary Material

Supplementary Figures

Supplementary Table 1

Supplementary Table 2

Supplementary Table 3

Supplementary Table 4

Supplementary Table 5

Supplementary Table 6

Supplementary Table 7

Supplementary Table 8

Supplementary Table 9

Supplementary Table 10

Supplementary Tables 11, 12 and 13
