# Supplementary material for: Identification of a ten-long noncoding RNA signature for predicting the survival and immune status of patients with bladder urothelial carcinoma based on the GEO database: a superior machine learning model
Source: Aging (Albany NY). 2021 Feb 17;13(5):6957–81. doi: 10.18632/aging.202553 (PMC7993680; doi:10.18632/aging.202553)
Supplement: Supplementary Figures [file aging-13-202553-s001.pdf]

SUPPLEMENTARY FIGURES

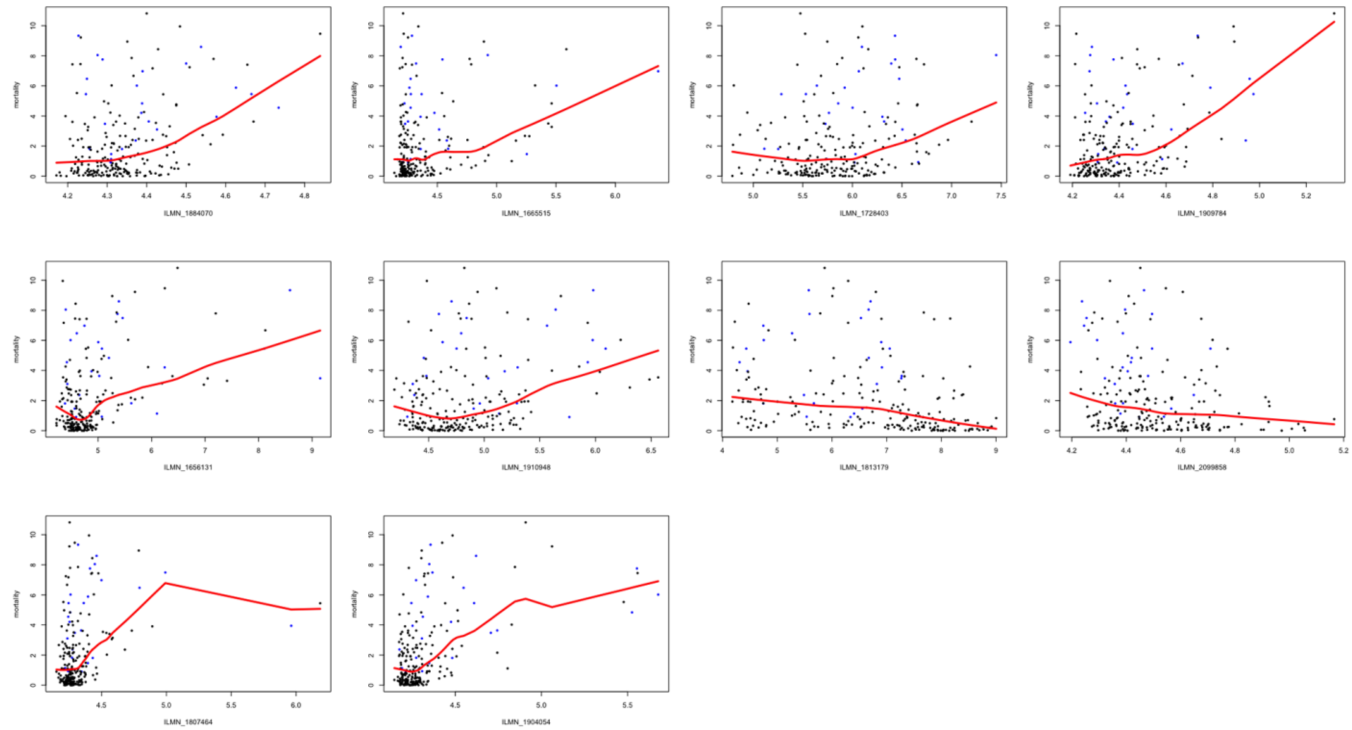

Supplementary Figure 1. Visualization of random survival forest-variable hunting analysis for identifying the ten most valuable prognostic lncRNAs.

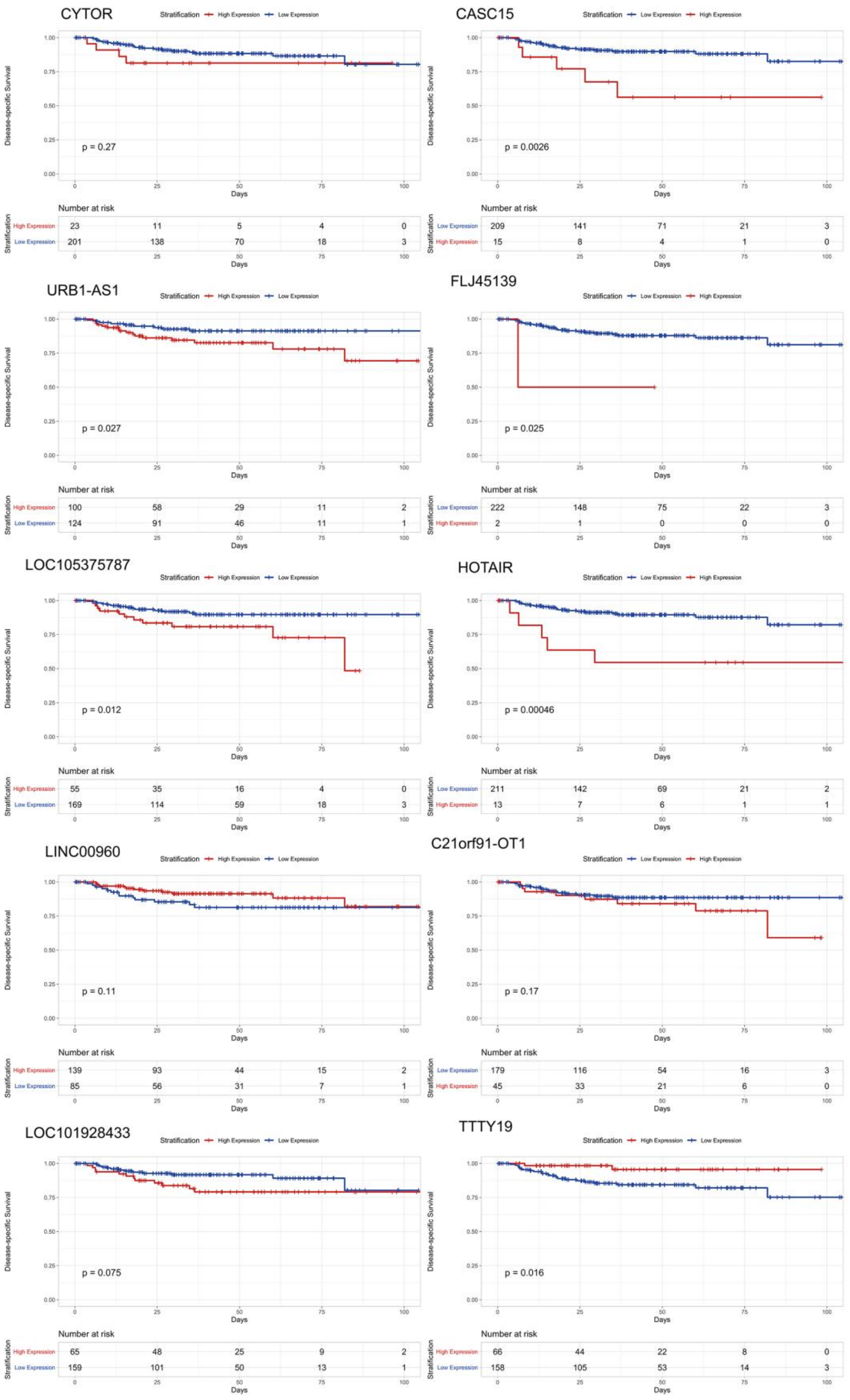

Supplementary Figure 2. Kaplan-Meier estimates of the patients in GSE32894. According to the ncRNA expression level, the patients were divided into two groups grouped using the k-means clustering algorithm.

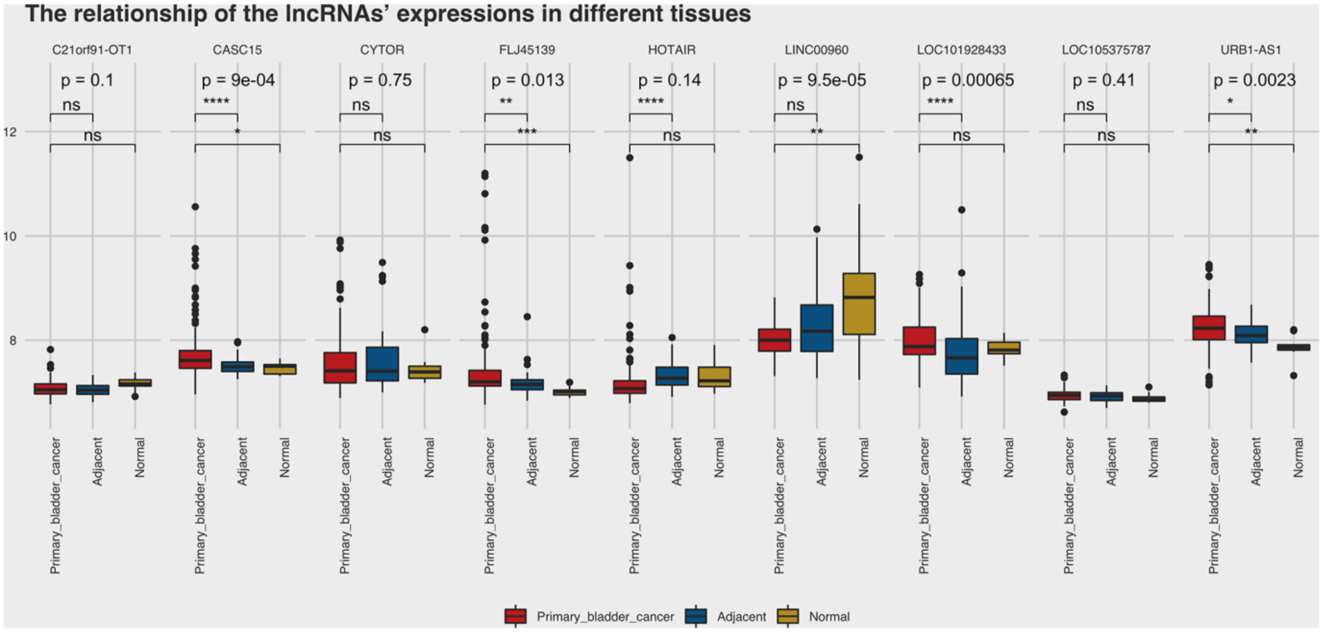

Supplementary Figure 3. Comparison of the expression levels of the 9 (9 out of 10) prognostic lncRNAs in BLCA tissue (n=165), cancer-adjacent tissue (n=58) and normal bladder mucosa (n=9) in GSE13507.
