# Supplementary material for: Identification of a ten-long noncoding RNA signature for predicting the survival and immune status of patients with bladder urothelial carcinoma based on the GEO database: a superior machine learning model
Source: Aging (Albany NY). 2021 Feb 17;13(5):6957–81. doi: 10.18632/aging.202553 (PMC7993680; doi:10.18632/aging.202553)
Supplement: Supplementary Tables 11, 12 and 13 [file aging-13-202553-s012.pdf]

SUPPLEMENTARY TABLES

Supplementary Table 11. Summarized information of the GEO datasets used in the study.

GEO number	Platform	Number of Sample	Date		Type of sample	Experiment type
			Submission	Last update		
GSE13507	GPL6102 Illumina human-6 v2.0 expression beadchip	165	Nov 07, 2008	Feb 03, 2020	primary bladder cancer	Expression profiling by array
GSE32548	GPL6947 Illumina HumanHT-12 V3.0 expression beadchip	131	Oct 11, 2011	Aug 16, 2018	urothelial carcinomas	Expression profiling by array
GSE32894	GPL6947 Illumina HumanHT-12 V3.0 expression beadchip	224	Oct 03, 2011	Aug 16, 2018	primary bladder cancer	Expression profiling by array

Supplementary Table 12. Condition number of a matrix (K) of the 10-lncRNA signature in the three data series.

Series	Condition Number of a Matrix(K)	If K<100
GSE32894	5.25	yes
GSE32548	2.69	yes
GSE13507	3.11	yes

If K < 100, the degree of collinearity is very small;
If 100 < = k < = 1000, there is a general degree of collinearity;
If k > 1000, there is a serious collinearity.

Supplementary Table 13. Results of univariate Cox regression analysis of the 10 prognostic lncRNAs in every series.

Univariate Cox proportional-hazards regression model analysis of DSS in patients with BLCA					
**Probe ID**	**Gene symbol**	**Beta**	**HR(95%CI for HR)**	**wald.test**	**p**
ILMN_1884070	LOC105375787	4.4	83 (5.2-1300)	9.8	0.0018
ILMN_1665515	CYTOR	1.2	3.4 (1.5-7.9)	8	0.0046
ILMN_1728403	URB1-AS1	1.2	3.5 (1.5-7.9)	8.8	0.003
ILMN_1909784	C21orf91-OT1	3.2	24 (3.3-170)	9.9	0.0016
ILMN_1656131	CASC15	0.62	1.9 (1.4-2.5)	16	7.60E-05
ILMN_1910948	LOC101928433	0.91	2.5 (1.3-4.9)	7	0.0083
ILMN_1813179	LINC00960	-0.39	0.68 (0.51-0.91)	6.7	0.0099
ILMN_2099858	TTTY19	-4.1	0.017 (0.00078-0.37)	6.7	0.0098
ILMN_1807464	FLJ45139	1.3	3.5 (1.5-8.4)	8.3	0.004
ILMN_1904054	HOTAIR	1.9	6.8 (2.9-16)	19	1.00E-05
**GSE13507**					
**Probe ID**	**Gene symbol**	**Beta**	**HR(95%CI for HR)**	**wald.test**	**p**
ILMN_1656131	CASC15	0.3	1.4 (0.79-2.3)	1.2	0.27
ILMN_1665515	CYTOR	0.84	2.3 (1.5-3.7)	12	0.00043
ILMN_1728403	URB1-AS1	0.75	2.1 (0.94-4.8)	3.3	0.069
ILMN_1807464	FLJ45139	0.16	1.2 (0.8-1.7)	0.69	0.41
ILMN_1813179	LINC00960	-0.47	0.62 (0.4-0.98)	4.2	0.039
ILMN_1884070	LOC105375787	0.76	2.1 (0.078-59)	0.2	0.65
ILMN_1904054	HOTAIR	0.048	1 (0.56-2)	0.02	0.88
ILMN_1909784	C21orf91-OT1	-0.86	0.42 (0.034-5.3)	0.44	0.51
ILMN_1910948	LOC101928433	-0.65	0.52 (0.23-1.2)	2.6	0.11
**GSE32548**					
**Probe ID**	**Gene symbol**	**Beta**	**HR(95%CI for HR)**	**wald.test**	**p**
ILMN_1884070	LOC105375787	2.8	16 (0.4-620)	2.2	0.14
ILMN_1665515	CYTOR	0.4	1.5 (0.45-5)	0.42	0.52
ILMN_1728403	URB1-AS1	0.65	1.9 (0.84-4.4)	2.4	0.12
ILMN_1909784	C21orf91-OT1	1.3	3.5 (0.5-25)	1.6	0.21
ILMN_1656131	CASC15	0.38	1.5 (1.1-2)	5.4	0.02
ILMN_1910948	LOC101928433	-0.49	0.61 (0.21-1.8)	0.82	0.36
ILMN_1807464	FLJ45139	-0.16	0.85 (0.11-6.6)	0.02	0.88
ILMN_1904054	HOTAIR	-0.81	0.44 (0.062-3.2)	0.65	0.42
ILMN_2099858	TTTY19	2	7.6 (0.25-230)	1.3	0.25
ILMN_1813179	LINC00960	-0.51	0.6 (0.43-0.84)	8.8	0.0031
